# Analysis of One Hundred Ophthalmic Cases, Showing the Comparative Frequency of the Various Diseases of the Eye, in Hamilton

**Published:** 1863-02

**Authors:** A. M. Rosebrugh

**Affiliations:** Hamilton, C. W.


*Analysis of one hundred Ophthalmic cases, showing the comparative frequency of the various diseases of the Eye, in Hamilton. Contributed by* A. M. ROSEBRUGH, M. D,, *Hamilton, C. W.*

Thinking it would be interesting to the readers of the *Journal,* I have transcribed from my case book the diagnosis of 100 cases of disease of the eye as they came under my observation during the months of March, April, May, June and July, 1862, in the city of Hamilton.

From observations made during a period of about three years, I am led to believe that the analysis of these 100 cases will give at least an approximate idea of the per centage in which the various diseases of the eye exist in Canada West.

| | |
|---|---|
| **DISEASES OF THE LIDS.** | |
| Entropion, | 1 |
| Ectropion, | 1 |
| Trichiasis, | 1 |
| Tinea, | 1 |
| Lippitudo, | 4 |
| Symblepharon, | 1 |
| Tumors, | 3 |
| **DISEASES OF THE LACHRYMAL ORGANS.** | |
| Strictures of the Canalicula, | 1 |
| Strictures of the Nasal Duct, | 1 |
| Mucocele, | 3 |
| **DISEASES OF THE EXTERNAL MUSCLES.** | |
| Strabismus, | 4 |
| Nystagmus, | 1 |
| Diplopia, | 1 |
| Paralysis of External Rectus, | 1 |
| Staphyloma, | 1 |
| **DISEASES OF THE IRIS.** | |
| Iritis—Idiopathic, | 1 |
| " Specific. | 1 |
| Synechia, | 2 |
| Occlusion of Pupil, | 2 |
| Prolapse of the Iris, | 1 |
| **DISEASE OF THE CHOROID.** | |
| Choroiditis, | 2 |
| Atrophy, (Amaurosis) | 3 |
| **DISEASE OF THE RETINA.** | |
| Retinitis, (Amaurosis) | 1 |
| Retinitis Pigmentosa, (Amaurosis) | 1 |
| Effusion upon Retina, (Amaurosis) | 1 |
| **DISEASE OF LENS AND CAPSULE.** | |
| Soft Cataract, | 1 |
| Hard Cataract, | 2 |

DISEASES OF THE COJUNCTIVA.

| | |
|---|---|
| Conjunctiva Simplex, | 3 |
| " Catarrhal, | 1 |
| " Phlyctenular, | 14 |
| " Granular, | 11 |
| " Graular with ulcers of Cornea, | 4 |
| Pterygium, | 2 |

DISEASE OF THE CORNEA.

| | |
|---|---|
| Nebulæ, (Scrofulous) | 2 |
| " (Syphilitic,) | 1 |
| Central Leucoma, | 3 |
| Pannus, | 2 |
| Keratitis, | 2 |
| Traumatic Cataract, | 1 |

DISEASES OF VITREOUS HUMOUR.

| | |
|---|---|
| Effusion in | 1 |

DISEASES OF THE OPTIC NERVE.

| | |
|---|---|
| Paralysis from cerebral injury. (Amaurosis) | 1 |

DISEASES OF ACCOMMODATION.

| | |
|---|---|
| Presbyopia, | 1 |
| Myopia, | 2 |
| Asthenopia, | 1 |

DILEASES OF THE BALL OF THE EYE.

| | |
|---|---|
| Ophthalmitis, | 1 |
| Atrophy, | 4 |

SUMMARY.

| | |
|---|---|
| Diseases of the Lids, | 12 |
| " " Lachrymal organs | 5 |
| " " External muscles, | 7 |
| " " Conjunctiva, | 35 |
| " " Cornea | 11 |
| " " Sclerotic, | 0 |
| " " Iris, | 7 |
| " " Choroid, | 5 |
| Disease of the Retina, | 3 |
| " " Lens and Capsule, | 4 |
| " " Vitreous Humour, | 1 |
| " " Optic Nerve, | 1 |
| " " Accommodation, | 4 |
| " " Ball of the eye, | 5 |
| " " Orbit, | 0 |
| Total, | 100 |

The proportion in which ophthalmic cases exist in proportion to a given population it would be difficult to ascertain; I will, however, venture the statement that they exist in this vicinity to the extent of about one per cent.

Hamilton, C.W., Sept. 12, 1862,

